# The functional architecture of S1 during touch observation described with 7 T fMRI

**DOI:** 10.1007/s00429-012-0489-z

**Published:** 2013-01-03

**Authors:** Esther Kuehn, Karsten Mueller, Robert Turner, Simone Schütz-Bosbach

**Affiliations:** Max Planck Institute for Human Cognitive and Brain Sciences, Stephanstr. 1a, 04103 Leipzig, Germany

**Keywords:** Empathy, Primary somatosensory cortex, Mirror neuron system, Shared representations, Suppressive interactions, Receptive field

## Abstract

Recent studies indicate that the primary somatosensory cortex (S1) is active not only when touch is physically perceived but also when it is merely observed to be experienced by another person. This social responsivity of S1 has important implications for our understanding of S1 functioning. However, S1 activity during touch observation has not been characterized in great detail to date. We focused on two features of the S1 functional architecture during touch observation, namely the topographical arrangement of index and middle finger receptive fields (RFs), and their dynamic shrinkage during concurrent activation. Both features have important implications for human behavior. We conducted two fMRI studies at 7 T, one where touch was physically perceived, and one where touch was observed. In the two experiments, participants either had their index finger and/or middle finger stimulated using paintbrushes, or just observed similar touch events on video. Our data show that observing and physically experiencing touch elicits overlapping activity changes in S1. In addition, observing touch to the index finger or the middle finger alone evoked topographically arranged activation foci in S1. Importantly, when co-activated, the index and middle finger RFs not only shrank during physical touch perception, but also during touch observation. Our data, therefore, indicate a similarity between the functional architecture of S1 during touch observation and physical touch perception with respect to single-digit topography and RF shrinkage. These results may allow the tentative conclusion that even primary somatosensory experiences, such as physical touch perception, can be shared amongst individuals.

**Electronic supplementary material:**

The online version of this article (doi:10.1007/s00429-012-0489-z) contains supplementary material, which is available to authorized users.

## Introduction

In recent years, the unisensory and private character of many primary sensory brain areas has increasingly been questioned. Traditionally, primary sensory brain areas, such as the primary somatosensory cortex (S1), have been assumed to be unisensory in character, and to only respond to modality-specific input; however, recent research has provided evidence for their multisensory response properties (Kayser 2010). The responsivity of primary sensory brain areas to multimodal input has even been called a “revolution in multisensory research” (Driver and Noesselt 2008), and S1 seems to be part of it. An increasing amount of evidence shows that S1 activity is not only influenced by direct somatosensory input, but is also modulated by other factors such as attention (Eimer et al. 2001; Hsiao et al. 1993; Macaluso et al. 2002), reward (Pleger et al. 2008), spatial processing (Eimer et al. 2001), or visual stimulation (Dionne et al. 2010; Zhou and Fuster 1997). As a consequence, the emerging view in cognitive neuroscience is that S1 can no longer be regarded as a strictly unisensory brain area, but rather as an area whose activity levels can be shaped by multiple environmental inputs.

Yet another quantum leap for our understanding of S1 is that recent studies have shown a specific influence of social cues on its functioning. Not only has evidence been provided that viewing the body compared with viewing an object can influence S1 processing during physical touch perception (Cardini et al. 2011; Fiorio and Haggard 2005; Longo et al. 2011), but it has also been shown that viewing touch to a body, without physically perceiving touch at all, can increase S1 activity levels (Blakemore et al. 2005; Ebisch et al. 2008; Kuehn et al. 2012; Schaefer et al. 2009, 2012). S1 activity changes during touch observation were shown to be stronger when human touch compared with object touch was observed (Blakemore et al. 2005), and a specifically *social responsivity* of S1 during touch observation has been assumed (Kuehn et al. 2012; Rossetti et al. 2012).

Importantly, active voxels in S1 that were triggered by touch observation were shown to overlap with voxels activated during physical touch perception (Blakemore et al. 2005; Ebisch et al. 2008; Schaefer et al. 2009). For example, using functional magnetic resonance imaging (fMRI), Blakemore and colleagues showed that somatotopically specific areas in S1 were activated when touch to another person’s face and neck was observed and that such activity changes overlapped with those areas that were activated during the physical perception of touch to the subject’s own neck and face (Blakemore et al. 2005). This is similar to the neuronal “resonance” responses reported for the motor system (Buccino et al. 2001; Gazzola and Keysers 2009; Mukamel et al. 2010), and the emotional system (Corradi-Dell’Acqua et al. 2011; Singer et al. 2004), where similar areas in the brain were shown to activate during action observation and execution, and emotion observation and perception, respectively. It is assumed that such resonance responses allow a basic understanding of the observed action or emotion, respectively (Bernhardt and Singer 2012; Fabbri-Destro and Rizzolatti 2008). According to this logic, S1 activity during touch observation should allow a basic understanding of another person’s somatosensory experiences as has recently been proposed (Avenanti et al. 2007; Keysers et al. 2010).

An interesting resulting question is how activity changes in S1 during touch observation can be characterized. Which features of the *functional architecture* of S1 are shared between physically perceiving and observing touch, and which only occur in one or the other condition? Based on recent neuroimaging research on the human somatosensory system, two features of the functional architecture of S1 seem to be of specific importance in this respect. On the one hand, the *topographical arrangement* of cortical receptive fields (RFs) in S1 plays an important role in our understanding of somatosensory processing. S1 activity during physical touch perception represents the contralateral side of the human body in a mediolateral sequence (Blankenburg et al. 2003; Gardner EK 2008; Kaas et al. 2002; Krause et al. 2001), and has been shown to make an important contribution to the ability to localize tactile stimuli on the skin (Beauchamp et al. 2009; Chen et al. 2003, 2007; Schweizer et al. 2001). Changes in the topographical arrangement of single-digit RFs in S1 can, for example, lead to a diminished ability to assign touch to a particular digit (Braun et al. 2000; Schweizer et al. 2001).

On the other hand, *suppressive interactions* between coactivated RFs in S1 have often been used to describe the functional architecture of S1. RFs in S1 react within milliseconds to sensory inputs given to surrounding RFs. For instance, the RF of one digit contracts when other digits are stimulated simultaneously and rapidly expands again when the digit is stimulated alone. This shrinking and re-enlarging of S1 RFs, depending on the tactile input given to surrounding RFs, has been demonstrated in studies on cats, mice, and monkeys (Friedman et al. 2008; Moore et al. 1999; Zarzecki and Wiggin 1982), and is posited to reflect a wide-spread cortical mechanism, which results in increased perceived stimulus contrast (Dykes 1983; Falkner et al. 2010; Jones 1993; Moore et al. 1999). Suppressive interactions during physical touch perception have also been investigated in multiple human studies (Biermann et al. 1998; Braun et al. 2002; Gandevia et al. 1983; Haavik Taylor and Murphy 2007; Hoechstetter et al. 2001; Ishibashi et al. 2000; Naka et al. 1998; Ruben et al. 2006; Tanosaki et al. 2002; Torquati et al. 2003), where the arithmetic sum of S1 signals measured during separate single-digit stimulations of two fingers was compared with the signal change during the stimulation of both fingers at once. In these studies, decreased signal strength during double-digit stimulation, compared with the sum of signals from separate single-digit stimulations, indicated how much the RFs contracted. Also in humans, such RF shrinkages presumably mediated by suppressive interactions, are assumed to positively relate to perceived stimulus contrast (Braun et al. 2002; Cardini et al. 2011; Puts et al. 2011).

Given that the aim of the present study was to characterize the functional architecture of S1 during touch observation along these two dimensions (topographical arrangement of RFs and suppressive interactions between RFs), the use of standard 3 Tesla (T) fMRI designs would have limited the scope of this approach. On the one hand, S1 activity during touch observation has been shown to be subtle, often only to survive significance thresholds when small volume corrections are applied to the fMRI data (Blakemore et al. 2005; Fitzgibbon et al. 2010; Keysers et al. 2004; Schaefer et al. 2009). This low signal strength clearly limits the possibility to characterize suppressive interactions, because subtle activity differences between different experimental conditions are of primary interest here. On the other hand, the spatial resolution of the functional data in 3 T fMRI studies is often too low to describe fine-grained architectonic characteristics in sufficient detail, for instance when specifying the topography of RFs in S1. In both cases, fMRI at ultra-high field offers a promising approach to circumvent some of these limitations (Chen et al. 2007; Kuehn et al. 2012; Stringer et al. 2011). As has recently been argued in a similar context (Kuehn et al. 2012; Stringer et al. 2011), 7 T fMRI can characterize brain activity changes in greater spatial detail, and with far greater sensitivity than is available with standard designs at 3 T (Bandettini 2009; Gati et al. 1997; Heidemann et al. 2012; Sanchez-Panchuelo et al. 2010; Scouten et al. 2006).

One recent study used 7 T fMRI to characterize S1 activity during touch observation (Kuehn et al. 2012). It was found that S1 activity during touch observation was restricted to posterior parts of contralateral S1; anterior parts were spared. This suggests that anterior S1 may still be considered a primary (and private) sensory brain area that is involved in social cognitive processes to a lesser extent, whereas somatosensory processes that are mediated by posterior S1 can be shared between, and are therefore influenced by, social interaction partners. However, that study did not characterize the functional architecture of activity changes in S1; it focused on the main effect of touch observation elicited by observing touch to one part of the hand. In order to characterize the topography of S1 activity during touch observation, one would have to let participants observe touch to different parts of the hands, such as different digits, and see whether distinct and topographically arranged activity changes in S1 were evoked. In addition, in order to characterize whether touch observation leads to similar inhibitory interactions between adjacent RFs, such as during physical touch perception (Gardner and Costanzo 1980; DiCarlo et al. 1998; DiCarlo and Johnson 2002; Friedman et al. 2008), one would have to compare the signal strength between conditions where touch to one finger is observed and conditions where touch to two fingers is observed, as is classically done in paradigms on suppressive interactions (Gandevia et al. 1983; Ruben et al. 2006).

For such an approach to be successful, it is important to know that single digits are distinctly represented not only in anterior S1, but also in posterior S1 (Duncan and Boynton 2007; Stringer et al. 2011; Sutherling et al. 1992), the area where touch observation elicits higher activity changes (Kuehn et al. 2012). More precisely, the RF of the index finger in posterior S1 is more lateral, more anterior, and more inferior than the RF of the middle finger of the same hand (Nelson and Chen 2008). A second important prerequisite for this approach is that, in principle, it has to be possible to characterize RF interactions by means of fMRI. Whereas many human studies have used EEG or MEG to characterize suppressive interactions between adjacently activated RFs in S1 (e.g., Biermann et al. 1998; Gandevia et al. 1983), one study with human participants successfully used fMRI for this purpose (Ruben et al. 2006). Importantly, fMRI was able to show that suppressive interactions between adjacently activated RFs in S1 occur not only in anterior but also in posterior parts of S1.

In the present study, we used fMRI at 7 T to characterize the functional architecture of S1 during touch observation along two dimensions: topographical arrangement of RFs and suppressive interactions between adjacent RFs. During the scanning session, participants observed video clips where touch was applied either to the index finger alone, the middle finger alone, or both the index and middle finger together using paintbrushes. While observing the videos, participants had to decide which of two subsequently presented paintbrushes offered the rougher stroke. This secondary task was included because the shrinkage of adjacent RFs during their concurrent activation is assumed to relate to more precise stimulus perception (Braun et al. 2002; Cardini et al. 2011; Puts et al. 2011), which we hypothesized could also transfer to the situation of touch observation. In this experiment (hereafter referred to as the observed touch experiment), no physical touch was applied to the participant’s hands or fingers. Based on previous findings (Kuehn et al. 2012), we expected that particularly posterior areas of contralateral S1 would show increased activity levels when touch videos were observed. Importantly, we further expected that activity foci during observed touch to the index finger alone and middle finger alone would be partly distinct and somatotopically ordered in accordance with the expected S1 functional topography. We also expected that observing touch to the middle finger and to the index finger together would decrease activity levels in S1 compared with observing touch to the same fingers alone. Such a reduction could likely be explained by suppressive interactions between adjacently activated RFs.

As a control experiment (hereafter referred to as the physical touch experiment), we performed another 7 T fMRI experiment where physical tactile stimulation was applied to participants’ index and middle fingers using paintbrushes in the same way as was observed in the observed touch experiment—the index finger alone, the middle finger alone, or both the index and middle fingers together. We expected the same pattern of results for this experiment, i.e., greater activity changes for physical touch perception compared with rest, partly distinct representations of the index and middle finger RFs in posterior S1, and suppressive interactions between index and middle finger RFs. In the conjunction analysis between both experiments, we expected an overlap between activity changes during physical touch perception and touch observation.

In summary, we intended to produce the first characterization of the functional architecture of S1 during touch observation, and relate the results to the well-described functional architecture of S1 during physical touch perception. Given the novelty of this experimental approach, our results should make an important contribution to understanding the role of S1 in social cognition.

## Materials and methods

### Experimental design

We conducted two separate fMRI experiments using a 7 T MR scanner, where participants either physically perceived tactile stimulation on their finger(s), or merely observed similar events on video. In the physical touch experiment, tactile stimulation was applied to participants’ index and/or middle finger using paintbrushes. In the observed touch experiment, videos were presented to participants showing touch to the corresponding fingers, again applied using paintbrushes. The observed touch experiment was always conducted first to exclude any influence of touch experience in the scanner on touch observation (Gazzola and Keysers 2009). Both experiments were separated by 6–7 days (mean: 6.5 days ± 1.1 days [SD]).

### Participants

Sixteen healthy volunteers between 22 and 30 years (mean age 25.6 years, 8 females) participated in our study. All were right-handed (mean handedness score on the Edinburgh inventory: 98.1; Oldfield 1971), had normal or corrected-to-normal vision and none reported history of neurological, major medical, or psychiatric disorders. They were paid for their attendance and informed consent was obtained from all participants. The study was approved by the local Ethics committee at the University of Leipzig. One participant was excluded from further analyses due to a high number of missed trials (9.4 %) and a reported failure to stay awake throughout the experiment. The functional and behavioral analyses were therefore conducted with data from 15 participants.

### Physical touch experiment

While subjects underwent fMRI scanning, physical tactile stimulation was applied via paintbrushes, either to the participants’ middle finger (MF) or index finger (IF), or simultaneously to both the middle and index fingers (both fingers = BF) of their right hands. In this way, the RF topography during physical touch perception could be described and later be compared to the RF topography during observation of touch to the corresponding fingers. In addition, added S1 activity changes during single-finger stimulation could later be compared with S1 activity changes during double-finger stimulation, as a measure of suppressive interactions (Gandevia et al. 1983; Ruben et al. 2006).

In the scanner, participants had their right hands fixed on a plastic board placed on the abdomen. The two paintbrushes used for tactile stimulation were mounted on two sticks that were connected to the plastic board to hold them at an optimal and fixed angle towards the fingers. To apply tactile stimulation, the two sticks were manually moved back and forth by the experimenter, who sat next to the scanner. Prior to the actual study, the experimenter was given intensive training in applying touch with constant pressure and with as little resultant movement as possible. Stimulation blocks lasted 24 s, consisted of four upward and four downward strokes (3 s per stroke), and were always followed by a 24 s rest period. In each run, the three stimulation blocks (IF, MF, BF) were repeated three times in a randomized sequence. The experiment consisted of four runs. Thus, each stimulation block was repeated 12 times throughout the experiment, which lasted about 30 min in total. Participants were instructed to close their eyes and to completely relax their hands and fingers throughout the scanning session. Trial order and temporal sequence of stimulation blocks were indicated to the experimenter via earphones.

### Observed touch experiment

**Fig. 1:**
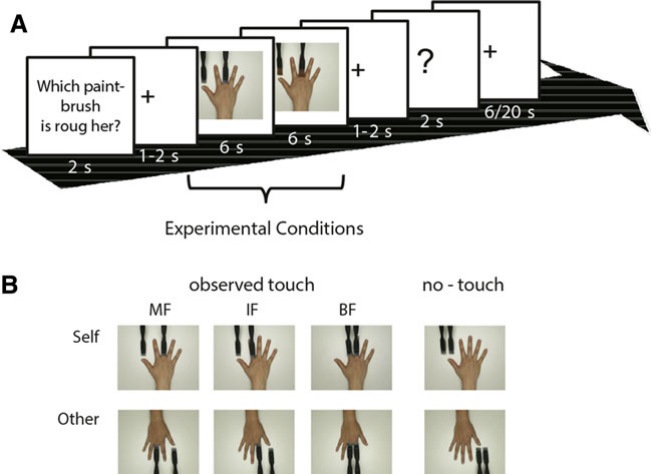
Design and example trial of the observed touch experiment. **a** Example trial as shown to participants in the scanner. Each trial started with the same question (“Which paintbrush is rougher?”) followed by two video clips presented in direct succession that belonged to the same experimental condition, but showed two different paintbrush pairs for tactile stimulation; while seeing a question mark on the screen, participants then had to indicate via left-hand button-presses which of the two paintbrush pairs was rougher. Half of the participants responded with their left index finger when they thought the first paintbrush pair was rougher and with their left middle finger when they thought the second was rougher, the other half responded vice versa; the pause between two trials was 6 s in two-thirds of the trials and 20 s in one-third of the trials, and this was counterbalanced across conditions. **b** Participants saw either their own hand in the first person perspective or another person’s hand in the third person perspective on video; in the observed touch conditions, touch was applied to either the middle finger (MF), the index finger (IF), or to both fingers (BF) of the right hand; in the no-touch condition, the paintbrush pair stroked the white surface on which the hand was positioned

During touch observation, participants in the scanner observed short video clips (6 s in duration) showing human right hands being touched by different paintbrushes. While watching the videos, no physical tactile stimulation was applied to the participants’ fingers. In the videos, analogous to the physical touch experiment, either the IF, MF, or BF of the right hand were stroked by one or two paintbrushes, respectively. When only one finger was stroked, the other paintbrush stroked the tabletop next to the hand. We also included a no-touch (control) condition, where both paintbrushes stroked the tabletop, while the hand was still visible. In half of the videos, participants saw their own hand from the first person perspective; in the other half they saw another person’s hand from the third person perspective. In this way, half of the observed touch and no-touch videos were related to the self, whereas the other half were clearly assigned to another person. Overall, this resulted in eight experimental conditions (observed touch IF/observed touch MF/observed touch BF/no-touch × self-related/other-related observed touch, see Fig. 1b). Throughout the experiment, each condition was repeated 12 times.

In each trial, two brief video clips were presented in direct succession that both belonged to the same experimental condition. In both clips, the paintbrushes shown differed in their roughness levels. After watching both video clips, participants had to indicate via left hand button presses whether they thought that the first or the second video displayed the rougher paintbrushes (see Fig. 1a for an example trial). A correct response could be given in each trial (see “Stimuli” for details). This secondary task was conducted to ensure constant attention during the experiment and to estimate how well participants could judge the sensory experience related to paintbrush strokes by sight.

### Design

The entire experiment took approximately 47 min in total and consisted of 96 trials. Half of the trials in each experimental condition showed the rougher paintbrushes first, the other half showed the smoother paintbrushes first. Trials were pseudo-randomly presented with the constraint that none of the experimental conditions was repeated more than twice in a row, and in such a way that trials in each condition added up to the same relative time point within the experiment. Prior to scanning, participants were allowed to practice the task in four trials outside the scanner room. The experimenter ensured that they understood the task well, and were familiar with the response mode prior to entering the scanner room.

### Stimuli

The video clips were recorded several weeks prior to scanning, with the same participants who later took part in the fMRI studies. During the video recordings, participants placed their right hand comfortably on a white table. Their right arm passed through a hole in a paper wall mounted in front of the table, so that they could not see their hand, the paintbrushes, or the experimenter throughout the video recordings. Their right hand was positioned such that their fingers did not touch each other but were also not stretched too far apart, and they were told to completely relax their right hand and fingers for the recording. For tactile stimulation, the experimenter used five different identical-looking paintbrushes [DaVinci paintbrushes, series 5025 (1), 5073 (2), 5036 (3), 5040 (4), and 5076 (5)]. Tactile stimulation was applied for 6 s, either to the participants’ right MF, IF or BF. In all conditions, two paintbrushes moved in parallel following a fixed temporal sequence that was indicated to the experimenter by an auditory signal (3 s per stroke, two strokes in total). All videos were recorded at a constant illumination level and with a constant angle and height between the video camera and the hands. During the video session, participants were blind with respect to the purpose of the video recordings, and they were not told how many or which kind of paintbrushes were used for stimulation.

During the fMRI experiment, the following paintbrush pairs were compared in the two videos of one trial: (1) versus (4), (2) versus (5), (1) versus (2), and (3) versus (5). Paintbrushes with higher numbers had softer and more flexible brushes and thus a smoother stroke than those with lower numbers. This was tested in a behavioral pre-experiment with an independent group of 9 participants. Roughness levels of all the paintbrush pairs used could be correctly distinguished well above chance (see Online Resource 1 for more information on this experiment). As a criterion to define self/other hand pairs, we matched the self-related and other-related hands for each participant in terms of gender, and chose partners with a similar hand shape index [i.e., ratio of width to length of hand (Longo and Haggard 2010); mean hand shape difference between self/other-pairs in our study: 4.4 %]. Importantly, once a self/other hand pair was identified, the videos that served as the self conditions for one partner always served as the other conditions for the matching partner. In this way, self/other differences in the video clips that could not be explicitly controlled (for example differences in skin color) were counterbalanced across participants.

### Imaging acquisition parameters

**Fig. 2:**
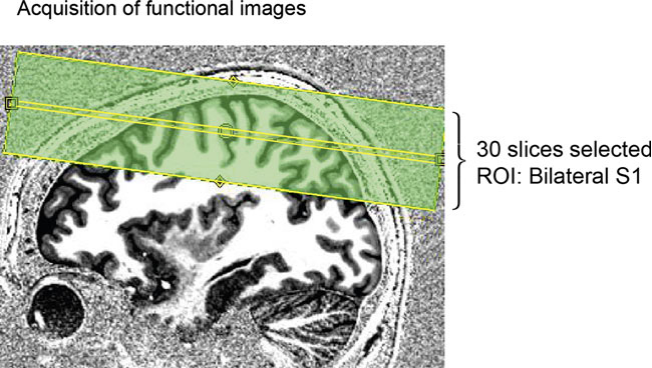
Selected slices for functional imaging of one example subject. Shown is a sagittal slice of the anatomical MP2RAGE scan which was used to select 30 axial slices covering bilateral S1 on the basis of the individual subject’s brain anatomy (i.e., hand knob area)

Functional and structural MRI data were acquired using a 7 T MR scanner (Magnetom 7T, Siemens Healthcare Sector, Erlangen, Germany) with a 24-channel NOVA head coil. Prior to both experiments, high-resolution 3D anatomical T1-weighted scans were acquired. For the observed touch experiment, which was always conducted first, structural data were acquired using the MP2RAGE sequence with the following parameters: TR = 5.0 s, TE = 2.45 ms, TI1/TI2 = 900 ms/2,750 ms, flip angle 1/flip angle 2 = 5°/3° with an isotropic voxel resolution of 0.7 mm (Marques et al. 2010). For the physical touch experiment, we used a shorter T1-sequence with a slightly reduced spatial resolution, because the T1-images acquired for each participant during the observed touch experiment could later be used for data analyses; the MP2RAGE sequence here served only to anatomically localize S1 in the subsequently measured functional scans. Acquisition parameters in the physical touch experiment were: TR = 4.0 s, TE = 2.36 ms, TI1/TI2 = 900 ms/2,750 ms, flip angle 1/flip angle 2 = 5°/3° with an isotropic voxel resolution of 0.9 mm. Shimming was performed in both experiments prior to collecting the functional data. In both experiments, T1-weighted scans were subsequently used to select 30 axial slices (interleaved slice acquisition, slice thickness = 1.5 mm, no gap) covering bilateral S1 and adjacent areas (see Fig. 2). The hand knob area was used for this purpose. This is easily identified in sagittal T1-images, and reliably indicates the location of the hand area in the primary motor cortex (Yousry et al. 1997), and the primary somatosensory cortex (Moore et al. 2000; Sastre-Janer et al. 1998; White et al. 1997). Functional T2*-weighted gradient-echo echo-planar images were then acquired using GRAPPA acceleration (iPAT = 3; Griswold et al. 2002). A field of view of 192 × 192 mm^2^ and an imaging matrix of 128 × 128 were used. The functional images had isotropic 1.5 mm voxels. The other sequence parameters were: TR = 1.5 s, TE = 20 ms, flip angle = 90°. The acquisition parameters of the functional scans were identical for the physical and observed touch experiments.

To attenuate scanner noise, participants were provided with earplugs and ear-defenders. During the observed touch experiment, the middle and index fingers of participants’ left hands were placed on two buttons of a response box. Visual stimuli were projected onto a plastic screen vertically mounted in front of the participants, which could be looked at via a mirror mounted on the receiver coil.

### fMRI preprocessing and localization of S1 activity

Preprocessing and statistical analyses of the functional imaging data were carried out using SPM8 (Statistic Parametric Mapping, Wellcome Department of Imaging Neuroscience, University College London, London, UK). A slice timing correction was applied to correct for differences in image acquisition time between slices, and realignment was performed to minimize movement artifacts in the time series (Unser et al. 1993a, b). Normalization to standard MNI space was done using the unified segmentation approach based on image registration and tissue classification (Ashburner and Friston 2005). The high-resolution T1-weighted images of both sessions were used to visually confirm correct registration between the sessions. In addition, the co-registration of each participant within the experiments was visually checked in order to identify possible spatial distortion effects that may occur at higher field strength (none were found, however). Data were filtered with a high-pass filter of 0.01 Hz to eliminate slow signal drifts. Data were smoothed with a Gaussian kernel of 4 mm full-width half-maximum (FWHM).

S1 is not a homogenous area, but can be classified into four sub-areas (from rostral to caudal: areas 3a, 3b, 1 and 2). The Anatomy Toolbox implemented in SPM8 (Eickhoff et al. 2005, 2006, 2007; Geyer et al. 1999, 2000; Grefkes et al. 2001) was used to specify in which sub-area activity changes took place for the normalized group-level and single-subject analyses. Single-subject analyses were additionally performed without normalization into stereotactic space in order to describe the site of the suppressive interaction effect as precisely as possible in relation to the cortical anatomy of each individual subject. We used guidelines that linked cytoarchitectonic labeling with anatomical descriptions of subregions (Geyer et al. 1999, 2000; Grefkes et al. 2001; White et al. 1997). According to these specifications, areas 3a and 3b (anterior S1) are found in the deep valley of the central sulcus and in the anterior wall of the postcentral gyrus, respectively, whereas areas 1 and 2 (posterior S1) are located at the crown of the postcentral gyrus and at the posterior wall of the postcentral gyrus, respectively. It is important to note that although these specifications are useful, no clear anatomical landmark exists for the exact transition zone between S1 subregions (Geyer et al. 1999). However, using a combination of automated and manual labeling, and by including single-subject analyses, the localization of the suppressive interaction effect in S1 in the current study is described in reasonable detail.

### fMRI statistical analyses

A general linear model (GLM) was fitted to the data and *t*-maps were created on the individual subject level. In the observed touch experiment, the observation times of the two video sequences in each trial were modeled as blocks and used to compute contrast images by linear combination of parameter estimates. In the physical touch experiment, the physical tactile stimulation blocks were used for this same purpose. In the observed touch experiment, the questions and the button-press events were included into the model as regressors. We used one-sample *t* tests at the second level to calculate different contrasts for the physical and observed touch experiments. All contrasts on the group level and on the normalized single-subject level were a priori masked with the anatomical S1 mask offered by the Anatomy Toolbox implemented in SPM8. Because the functionally scanned regions were manually selected in each individual subject based on the T1 scans (see Fig. 2), thus always covering S1 but covering varying parts of the motor or parietal cortices depending on the individual subject’s brain anatomy, this anatomical masking ensured comparable data analyses for all participants.

For the group-level calculations, reported voxels of the functional data were considered significant at *p* < 0.001 when belonging to a cluster significant at *p* < 0.05 (FWE-corrected). In addition, we describe some of the group-level clusters at uncorrected cluster-thresholds (i.e., without FWE-correction) when this provided additional information (for example on the question of whether ipsilateral S1 would show any sub-threshold activity). When these more liberal analyses were used, it is explicitly pointed out in the results section. In addition to group-level analyses, single-subject analyses were also performed in order to describe some of the reported effects in greater detail. For the single-subject analyses, reported voxels were thresholded at *p* < 0.001. They are mentioned when belonging to a cluster with a minimum size of five voxels (16.8 mm^3^).

#### Topographical arrangement

For the physical touch experiment, we calculated the main effect of physical touch perception versus rest (IF touch + MF touch + BF touch − 3 × rest), and the specific effects of touch applied only to the IF, MF, and to BF, respectively (e.g., IF touch – rest). For the observed touch experiment, we calculated the main effect of observing touch to the hand versus observing no touch to the hand, as presented in the videos [(self-related observed touch IF + self-related observed touch MF + self-related observed touch BF − 3 × no-touch self) + (other-related observed touch IF + other-related observed touch MF + other-related observed touch BF − 3 × no-touch other)]. This effect was also calculated separately for observing touch to the IF, MF and BF, respectively. To look at whether S1 RFs during observed touch were topographically aligned, we masked the observed touch contrasts with the corresponding physical touch masks (e.g., observed touch IF – no-touch masked with IF touch – rest) and non-corresponding physical touch masks (e.g., observed touch IF – no-touch masked with MF touch – rest). Physical touch masks contained all voxels significant at *p* < 0.001 that belonged to a cluster significant at *p* < 0.05 (FWE-corrected). In addition, we estimated whether the topographical arrangement of S1 RFs during observed touch followed the expected pattern (i.e., the RF of the IF was supposed to be more lateral, more anterior, and more inferior than the RF of the MF).

#### Suppressive interactions

To calculate suppressive interactions between adjacent RFs in S1, we compared the expected activity changes to the actual activity changes during BF physical stimulation or observation, respectively. More precisely, for the physical touch experiment, we calculated the expected activity changes in S1 in the case where signal changes during touch applied to the IF and MF added up linearly. This was calculated as a first-level contrast [i.e., (IF touch – rest) + (MF touch – rest)]. This contrast was then compared with the actual activity changes during BF stimulation (i.e., BF touch – rest). Also this calculation was performed at the first-level. Note that this is analogous to how suppressive interactions during physical touch were characterized in a previous fMRI study (Ruben et al. 2006), as we confirmed in an additional analysis (results not reported).

For the observed touch experiment, analogous methodology was applied: to measure suppressive interactions during observed touch, we first calculated the expected activity changes in S1 if activity changes during observed touch to the IF and MF added up linearly [i.e., (self-related observed touch MF + self-related observed touch IF − 2 × no-touch self) + (other-related observed touch MF + other-related observed touch IF − 2 × no-touch other)]. To define suppressive interactions during observed touch, this contrast was compared with actual S1 activity changes during observed touch to BF [i.e., (self-related observed touch BF − no-touch self) + (other-related observed touch BF − no-touch other)]. These same analyses were also performed separately for the self- and other-related observed touch conditions.

The suppressive interaction contrast therefore specified signal decreases in S1 that particularly occur in the BF stimulation and observation conditions, respectively. Signal decreases in these conditions that most likely reflect suppressive interactions are those which occur in voxels that belong to the RF of one of the two single fingers. Other signal drops can be less easily explained by suppressive interactions between adjacently activated RFs. We, therefore, spatially specified the suppressive interaction contrast by only describing significant activity decreases in voxels that that belonged to the RFs either of the IF or of the MF. More precisely, the suppressive interaction contrast for physical touch perception was masked by all voxels that were activated by physical IF stimulation plus those that were activated by physical MF stimulation at *p* < 0.001 belonging to a cluster of *p* < 0.05 (FWE-corrected; see Ruben et al. 2006 for a similar approach). The suppressive interaction effect for observed touch was analogously masked by all voxels that were activated during observed touch to the IF plus those that were activated during observed touch to the MF. Here, we restricted our search volume to this mask area. Note that this spatial specification was necessary in order to clearly identify signal decreases that were specific to the effect under investigation (i.e., suppressive interactions).

We also needed a way of quantifying the degree of suppressive interactions, both during physical touch perception and during touch observation. Using contrast estimates, we calculated interaction ratios (IRs), which have frequently been used to specify the relation between expected and real activity changes in S1 (IR = 100 − ([BF/(IF + MF)] × 100); Biermann et al. 1998; Hsieh et al. 1995; Ishibashi et al. 2000; Ruben et al. 2006). We used the masks created for specifying the suppressive interaction contrast for physical touch and observed touch (see previous paragraph) to extract all relevant contrast estimates for each individual subject. The individual IRs could then be used to calculate the mean IR across participants. This allows a much more detailed and spatially specific analysis than taking the contrast estimates as a mean across the whole group of participants. However, this procedure comes with the cost of only allowing the analysis of those participants for whom a mask could be created. More precisely, only those participants who showed significant activity changes at the single-subject level for physically/visually perceiving touch to the MF and IF could be included. As expected, this was the case for all participants with respect to physical touch perception (*N* = 15). However, this was not the case for all participants with respect to touch observation. More precisely, *n* = 10 participants could be used to calculate the mean IR for observed touch, and *n* = 9 participants could be used to calculate the mean IR for self-related observed touch. For other-related observed touch, only *n* = 4 participants fulfilled these criteria (i.e., *n* = 11 participants did not show significant activity changes when observing other-related touch to the MF or to the IF at the single-subject level). Thus, the mean IR for other-related observed touch was not calculated.

To summarize, whereas voxel-wise statistics included all participants (*N* = 15), contrast estimates to calculate the IR during observed touch could only be extracted for a subset of participants due to the masking procedure that required significant single-subject results [*n* = 10 for main effect of observed touch, *n* = 9 for self-related observed touch, *n* = 4 for other-related observed touch (not calculated)].

#### Overlapping RFs

To find out whether suppressive interactions occurred only in voxels where IF and MF RFs overlapped (and could, thus, theoretically be explained by ceiling effects of the BOLD signal, for example), or also occurred in non-overlapping voxels, we additionally calculated whether suppressive interactions occurred only in “overlapping”, or also in “non-overlapping” S1 voxels. Overlapping voxels were defined as those voxels in S1 that were active when both touch to the IF and touch to the MF were observed (i.e., observed touch IF – no-touch ∩ observed touch MF – no-touch) or experienced (IF touch – rest ∩ MF touch – rest), whereas non-overlapping voxels were those which did not overlap between the two contrasts. We calculated the percentage (%) of suppressive interaction voxels in either category (i.e., suppressive interaction effect in overlapping and non-overlapping voxels) separately for physical and observed touch.

### Behavioral analyses

During the observed touch experiment, participants solved a secondary two-alternative forced-choice task in the scanner, in which they had to indicate which of two subsequently presented video clips displayed the rougher paintbrush pair. The two video clips presented in one trial always showed paintbrushes of different roughness levels, such that a correct or incorrect response could be given in each trial. We performed a repeated-measures analysis of variance (ANOVA) to estimate the influence of hand identity (self-related, other-related) and observed event (observed touch, no-touch) on the percentage of correct responses given in this task. We also calculated a one-way ANOVA to estimate the influence of finger touch (MF, IF, BF) on the percentage of correct responses. To investigate whether the individual degrees of suppressive interactions across trials were related to how precisely roughness levels could be estimated by sight, we performed Pearson correlations between individual IRs during touch observation and the percentage of correct responses both for the IRs across conditions and the IRs for the self-conditions.

## Results

### Topographical arrangement

**Fig. 3:**
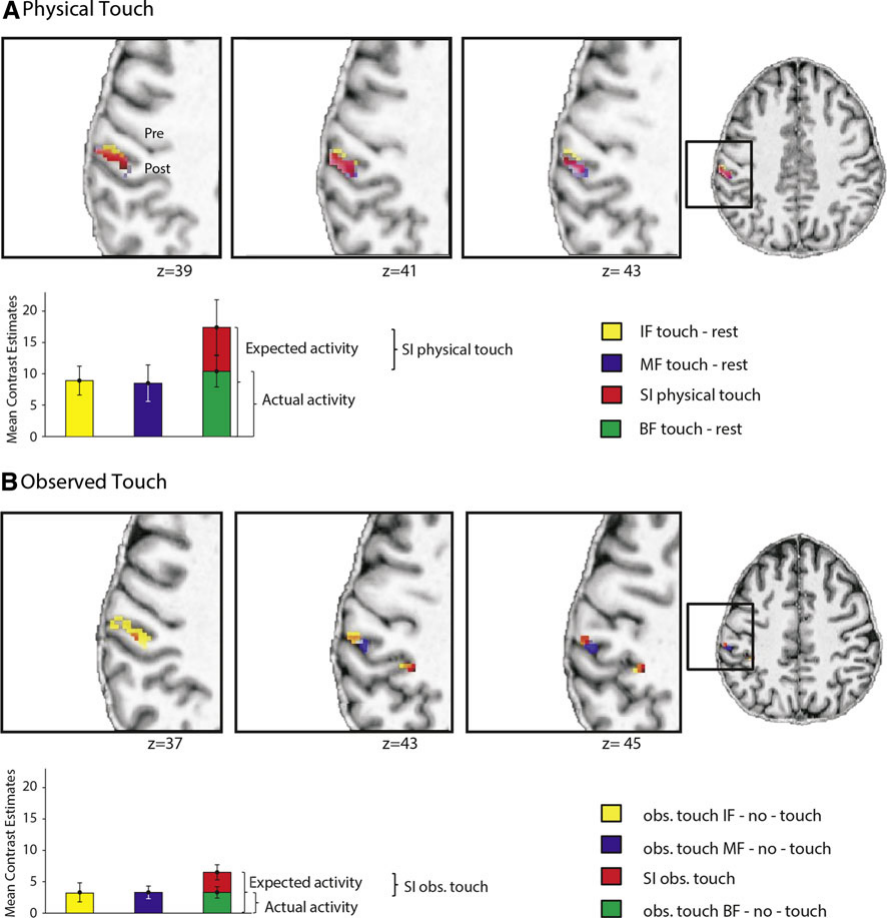
Suppressive interactions (SI) in contralateral S1 during physical touch perception (**a**) and touch observation (**b**). **a** Activity changes of contralateral S1 during physical touch applied to the index finger (IF) and the middle finger (MF); in addition, the suppressive interaction (SI) effect for physical touch is displayed using voxel-wise statistics in the* upper panel* (IF touch–rest + MF touch–rest − BF touch–rest), and using contrast estimates in the *lower panel*; the *bar* labeled “Expected activity” describes the added contrast estimates of physical touch to the IF and MF, whereas the *bar* labeled “Actual activity” describes the contrast estimates when both fingers were stimulated together; the *bar graphs* show mean contrast estimates ± standard deviation (SD) of all (*N* = 15) participants. **b** Activity changes of contralateral S1 during observed touch to the IF and MF; in addition, the SI effect for observed touch is displayed using voxel-wise statistics in the *upper panel* (obs. touch IF–no-touch + obs. touch MF–no-touch − obs. touch BF–no-touch), and using contrast estimates in the *lower panel*; the *bar* labeled “Expected activity” describes the added contrast estimates of touch observation to the IF and MF, whereas the *bar* labeled “Actual activity” describes the contrast estimates when touch to both fingers together was observed; the *bar graphs* show mean contrast estimates ± standard deviation (SD) of *n* = 10 participants (see “Suppressive interaction” for details on why not all participants were part of this analysis); functional images are masked with an anatomical mask covering contralateral S1 and are thresholded at *p* < 0.0005 (uncorrected) (**a**) and *p* < 0.001 (uncorrected) (**b**); the data are displayed on a normalized T1-image of an individual subject; *Pre* precentral gyrus, *Post* postcentral gyrus

As expected, physical touch administered to participants’ right fingers activated, as a main effect, a large significant cluster in left (contralateral) S1 that peaked in left posterior S1, and extended to left anterior S1. Touch applied specifically to the right IF, MF, or to BF, respectively, also activated significant focal areas in left S1. The IF and MF RFs partly overlapped, but were also partly distinct. The significant clusters peaked in left posterior S1, but extended to left anterior S1 (see Fig. 3a; Table 1). No significant activity changes were found in right (ipsilateral) S1 for these contrasts. We also looked at sub-threshold activity in ipsilateral S1. Here, we found that when the significance threshold of *p* < 0.001 and *k* ≥ 5 was not FWE-corrected, one cluster in right (ipsilateral) S1 showed greater activity during physical touch perception compared with rest [*k* = 9; *t* = 4.05; 53, −18, 34 (*x*, *y*, *z*)] (see Online Resource 2 for a complete list of sub-threshold activity changes).

**Table 1:** S1 activity changes during physical touch perception and touch observation in different experimental conditions

Contrast	Area	MNI location (*x*, *y*, *z*)	Peak *t* value	No. of voxels
Physical touch				
MF touch + IF touch + BF touch–rest	L Area 2	−55 −24 40	8.31	685
MF touch–rest	L Area 2	−55 −24 40	7.33	185
	L Area 2	−44 −33 57	6.78	215
IF touch–rest	L Area 2	−37 −39 63	9.03	562
BF touch–rest	L Area 2	−56 −22 42	9.37	991
SI physical touch	L Area 2/L IPC	−54 −26 39	7.47	123
Observed touch				
Obs. touch MF + obs. touch IF + obs. touch BF − no-touch	L Area 2	−37 −44 54	4.35	245
	L Area 2	−55 −24 44	4.04	179
# Obs. touch MF − no-touch	L Area 1	−26 −54 66	5.02	6
	L Area 2	−26 −51 57	5.00	17
	L Area 2	−54 −27 45	3.45	19
	L Area 1	−60 −18 36	3.29	9
Obs. touch IF − no-touch	L Area 2	−58 −21 40	6.05	139
Obs. touch BF − no-touch	L Area 2	−34 −44 57	6.27	127
	L Area 2	−54 −24 44	5.56	128
SI observed touch	L Area 2	−55 −26 46	4.54	8
	L Area 2	−36 −36 44	4.51	5
SI observed touch self	L Area 2	−40 −42 62	4.36	9
Observed touch ∩ Physical touch				
Obs. touch MF + obs. touch IF + obs. touch BF − no-touch ∩ MF touch + IF touch + BF touch–rest	L Area 2	−55 −24 44	5.70	162
# Obs. touch MF–no-touch ∩ MF touch–rest	L Area 2	−54 −27 45	4.45	15
	L Area 1	−61 −16 33	4.15	10
Obs. touch IF–no-touch ∩ IF touch–rest	L Area 2	−58 −21 40	6.05	123
Obs. touch BF–no-touch ∩ BF touch–rest	L Area 2	−54 −24 44	5.56	114

Listed clusters contain voxels thresholded at *p* < 0.001 and are cluster-corrected at *p* < 0.05 (FWE-corrected); the two contrasts which are marked with a # show clusters that are *not* cluster-corrected, but contain a minimum of five voxels using the same voxel threshold; see Online Resource 2 for a complete list of sub-threshold activity changes for all listed contrasts

*obs.* observed, *MF* middle finger, *IF* index finger, *BF* both fingers, *SI* suppressive interactions, *IPC* inferior parietal cortex

For observed touch, we found that looking at a hand being touched compared with looking at the same hand not being touched significantly increased activity in left (contralateral) posterior S1. Note that participants did not receive any tactile stimulation in either of these observation conditions. No significant activity changes were found for the reverse contrast (no-touch vs. observed touch). In addition, we were interested in whether right (ipsilateral) S1 would show any sub-threshold activity during touch observation. When we omitted the FWE-correction, significant activity changes in ipsilateral S1 were found (*p* < 0.001 and *k* ≥ 5). This cluster was localized in posterior parts of right S1 (see Online Resource 2 for a list of all sub-threshold activity changes).

Observing touch to specific fingers also activated left (contralateral) S1. Whereas activity changes in left S1 during observed touch to the IF and to BF survived the standard cluster-corrected thresholds, activity change in left S1 during observed touch to the MF was only significant when no cluster-correction was applied (*p* < 0.001 and *k* ≥ 5). All observed touch clusters peaked in left posterior S1 (see Table 1; Fig. 5). To verify that activity changes in contralateral S1 in response to touch observation were restricted to posterior S1, and did not occur in anterior S1 (particularly in area 3b), we conducted an ROI analysis focusing on left area 3b. We masked the contrast observed touch–no-touch with the left area 3b mask provided by the Anatomy toolbox implemented in SPM. Here, we found that no significant activity changes survived the standard significance threshold, even when voxels at *p* < 0.001 and *k* ≥ 5 belonging to uncorrected clusters were taken into account.

**Fig. 4:**
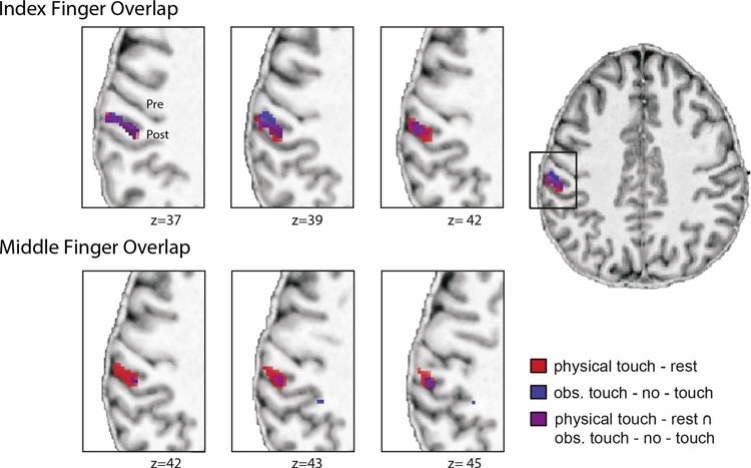
Overlap between activity changes during physical touch perception and touch observation in contralateral S1. Overlapping voxels between physical touch perception and touch observation are displayed in purple; overlaps are shown separately for the index finger (*upper panel*) and the middle finger (*lower panel*); functional images are masked with an anatomical mask covering contralateral S1 and thresholded at *p* < 0.001 (uncorrected); functional data are visualized on a normalized T1 image of an individual subject; *Pre* precentral gyrus, *Post* postcentral gyrus

We also looked at whether S1 activity changes during observed touch overlapped with S1 activity changes during physical touch. We masked the contrast observed touch–no-touch with physical touch–rest, and found that significant clusters for touch observation were present in posterior contralateral S1. Similarly, we found that activity changes specific to observing touch to the IF were still significant when masked with the effect of physically experiencing touch to the IF (number of voxels: 124), but not when masked with the effect of physically experiencing touch to the MF (number of voxels: 111). The significant overlap was found in left posterior S1 (see Table 1; Fig. 4). As explained above, observing touch to the MF evoked activity changes in contralateral S1, significant only when no cluster-correction was applied. We then looked at whether these clusters would be preserved when masked with physical touch to the IF or MF. We found that one cluster remained significant both when masked with physical touch to the MF and when masked with physical touch to the IF (number of voxels MF mask: 25, number of voxels IF mask: 23). This overlap was also found in left posterior S1 (see Table 1; Fig. 4). To estimate whether the mask would have any significant effect on the number of voxels included in the corresponding and non-corresponding masks, we performed a Chi-square test including the number of voxels significant within the two mask conditions of both contrasts. This test did not reach statistical significance (*p* > 0.2). When estimating the percentage of participants who showed overlapping activity changes between physical and observed touch in the different conditions, we found that 75 % of participants showed shared voxels for observed touch to the IF, 78 % showed shared voxels for observed touch to the MF, and 92 % showed shared voxels for observed touch to BF.

**Fig. 5:**
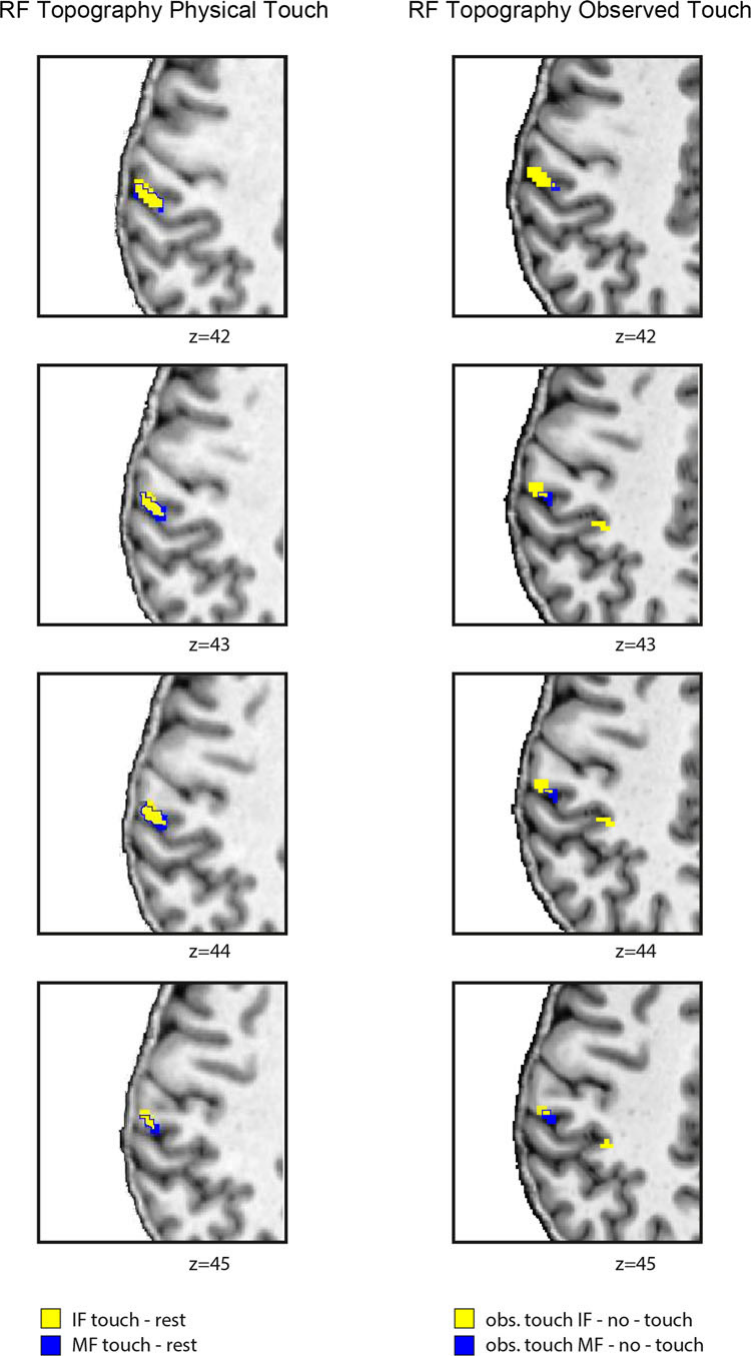
Receptive field (RF) topography of the index finger (IF) and middle finger (MF) in contralateral S1 during physical touch perception and touch observation. Shown are five axial slices ordered from inferior (*z* = 42) to superior (*z* = 47) of *N* = 15 participants; the borders of the MF RFs are indicated using *blue lines*; functional images are masked with an anatomical mask covering contralateral S1 and visualized at an individual’s normalized T1 image; to make both conditions better comparable, a slightly more conservative threshold was chosen for physical touch perception [*p* < 0.0001 (uncorrected)] than for touch observation [*p* < 0.001 (uncorrected)]

We additionally looked at the topographic arrangement of activity changes as evoked by observing touch to the IF and the MF, respectively. We found that activity changes in both conditions were partly overlapping, but partly distinct. Importantly, activity changes evoked by observing touch to the IF were more lateral, more anterior, and more inferior than activity changes evoked by observing touch to the MF (see Table 1; Fig. 5).

One last analysis was performed due to a concern that activity changes in contralateral S1 during touch observation could be explained by preparatory motor activity for the later button-press responses rather than by touch observation. To counter this argument, we looked at whether left and right primary motor cortex (M1) showed any increased activity changes during touch observation that could indicate preparatory motor activity or motor imaginary during touch observation. There were no significant activity changes neither in left nor in right M1 for the observed touch–no-touch contrast. This was also true when not correcting for multiple comparisons.

### Suppressive interactions

**Fig. 6:**
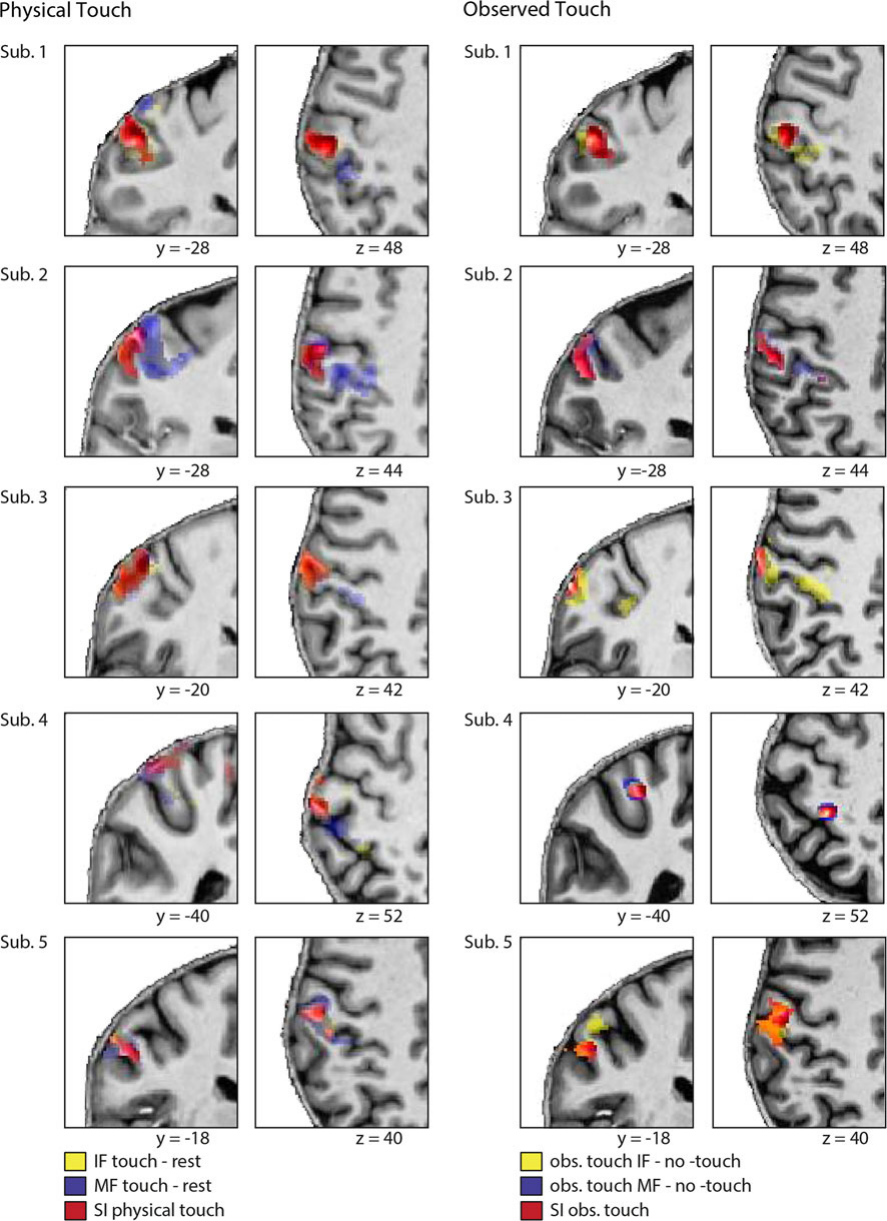
Contralateral S1 activity during physical touch perception and touch observation of *n* = 5 individual subjects. The *left side* of the figure shows functional data of physical touch to the index finger (IF), the middle finger (MF), and the suppressive interaction (SI) effect for physical touch; the *right side* of the figure shows functional data of observed touch to the IF, the MF, and the SI effect for observed touch; note that the same axial and coronal slices of the same subjects can here be visually compared; functional data are presented at the individual’s normalized T1-anatomical scans; to make both conditions better comparable, a slightly more conservative threshold was chosen for physical touch perception [*p* < 0.001 (uncorrected)] than for touch observation [*p* < 0.005 (uncorrected)]

In order to estimate the degree of suppressive interactions in S1 during physical touch perception, we compared the summed activity changes during physical touch perception to the IF and MF to the activity changes in the BF stimulation condition. We found a significant suppressive interaction effect for physical touch in left (contralateral) S1, which peaked at the border between left area 2 and the left inferior parietal cortex, and extended to left area 2, left BA 1, and left area 3b (see Table 1; Fig. 3a). No significant suppressive interaction effect was found in right (ipsilateral) S1, even when the analysis was performed without correcting for multiple comparisons. At the individual subject level, suppressive interactions for physical touch were significant in all but one of the investigated participants (*n* = 14), and could be assigned to left area 1 and left area 2. Only a subset of participants showed additional significant activity changes in left area 3b (*n* = 4) and left area 3a (*n* = 2) for this contrast. The mean IR for physical touch perception was 37.2 %, SD = 15.9 (Fig. 3a, see Fig. 6 for single subject data).

We then calculated the suppressive interaction effect for observed touch, which was similarly calculated by comparing summed activity changes in S1 evoked by observing touch to two single fingers separately to activity changes evoked by observing touch to both fingers together. Here, we found a significant suppressive interaction effect in two clusters that both peaked in left (contralateral) area 2. One cluster also extended to left area 1, the other extended to the left superior parietal lobule (see Table 1; Fig. 3b). No significant suppressive interaction effect was found for right (ipsilateral) S1. At the individual subject level, we found a significant suppressive interaction effect in left S1 for *n* = 8 participants (see individual subject data of *n* = 5 participants in Fig. 6). In all of them, the effect was located in left area 2. In *n* = 7 participants, activity changes also extended to left area 1, and in one subject, the activity changes extended to left area 3. Note that most of the other participants also showed suppressive interaction voxels in left S1 during observed touch, but these results are not reported due to the relatively conservative single-subject threshold we defined for our analyses [e.g., *n* = 14 participants showed a suppressive interaction effect when we lower the single-subject threshold to *p* < 0.005 (uncorrected)].

We also calculated the suppressive interaction effect specifically for self- and other-related observed touch. The suppressive interaction effect for self-related observed touch revealed one significant cluster in left area 2. The suppressive interaction effect for other-related observed touch did not reveal any significant activity changes in S1. At the individual subject level, *n* = 8 participants showed a suppressive interaction effect for self-related observed touch, and *n* = 4 participants showed a suppressive interaction effect for other-related observed touch. The mean IR for self-related observed touch was 54.93 %, SD = 11.35. The mean IR for observed touch (main effect) was 50.22 %, SD = 9.35.

### The role of overlapping RFs

We found that the suppressive interaction effect for physical touch occurred in both overlapping and non-overlapping S1 voxels. More precisely, we found part of the cluster of the suppressive interaction effect for physical touch in overlapping voxels [*k* = 113; *t* = 7.47; −54, −26, 39 (*x*, *y*, *z*), localized in left area 2, the left inferior parietal cortex, and left area 1] and part of the cluster in non-overlapping voxels [*k* = 7, *t* = 4.74, −52, −21, 38 (*x*, *y*, *z*), localized in left area 2, the left inferior parietal cortex, and left area 3b]. Also at the individual subject level, the suppressive interaction effect for physical touch occurred within both overlapping and non-overlapping S1 voxels in all subjects. As a mean across participants, 79 % of the suppressive interaction voxels were overlapping voxels, whereas 21 % were non-overlapping voxels.

The suppressive interaction effect for observed touch was, on the group level, only observed in non-overlapping S1 voxels [*k* = 14; *t* = 4.61; −56, −24, 45 (*x*, *y*, *z*), localized in left area 2]. When looking at the individual subject level, however, the effect was found both in overlapping and non-overlapping S1 voxels in all participants. Across participants, 60 % of the voxels that showed a suppressive interaction effect were overlapping voxels, and the remaining 40 % were non-overlapping voxels. For self-related observed touch, again suppressive interactions at the group level were only found in non-overlapping S1 voxels [*k* = 14; *t* = 4.62; −42, −40, 62 (*x*, *y*, *z*), localized in left area 2, *k* = 9; *t* = 4.93; −49, −36, 57 (*x*, *y*, *z*)]. At the single subject level, however, the effect was again found in both overlapping and non-overlapping S1 voxels. Here, 56 % of the voxels that showed suppressive interactions for self-related observed touch were overlapping voxels, and the remaining 44 % were non-overlapping voxels.

### Behavioral results

All participants performed the visual roughness discrimination task (where participants had to distinguish between roughness levels of different paintbrush pairs by sight) with high levels of accuracy [self touch: 93.1 % ± 5.4 (SD), self no-touch: 95.0 % ± 7.6 (SD), other touch: 91.8 % ± 7.4 (SD), other no-touch: 91.7 % ± 7.0 (SD), *N* = 15]. The participants maximally missed two trials throughout the entire experiment. The percentage of correct responses in the visual roughness discrimination task was not significantly influenced by hand identity (self, other) or the presence of hand touch (observed touch, no-touch). There was also no significant interaction between these two factors (*p* > 0.1). Also, the finger that was touched (IF, MF, BF) did not influence the percentage of correct responses, neither across conditions nor for the self- and other-related conditions separately (*p* > 0.05). With respect to how individual IRs related to the degree to which roughness levels could be distinguished by sight, there was a significant correlation between the individual IRs when the self was observed and the accuracy to solve the visual roughness discrimination task when self-related touch to the IF and to BF was observed (*r* = 0.78 for IF, and *r* = 0.76 for BF self, *p* < 0.05, two-tailed, see Online Resource 3). There was no such relation between the individual IRs during observed touch and percentage of accuracy across conditions. Note, however, that the number of subjects whose data were available to calculate this correlation (*n* = 9) was very small, such that this positive relation between individual IRs and behavioral performance has to be replicated and verified by future studies using a greater number of participants.

## Discussion

The present study offers the first detailed characterization of the functional architecture of S1 during touch observation. Our data show that posterior parts of contralateral S1 in particular, but not anterior parts, are activated when touch is observed on video. Activity changes in posterior S1 elicited by touch observation also overlap with those elicited by physical touch perception. Importantly, observing touch to the index finger alone or the middle finger alone offers a similar topographical arrangement of RFs in S1 as those elicited by physically perceiving touch to the same fingers. In addition, index and middle finger RFs show the characteristic dynamic shrinkage when activated concurrently not only during physical but also during visual touch perception. Our study, therefore, provides novel evidence indicating that the functional architecture in posterior S1 with respect to RF topography and RF interaction is similar between touch observation and physical touch perception.

### Posterior S1 activity during touch observation

In the present study, short video clips were presented to participants, which showed right hands being touched or not being touched by paintbrushes. By comparing observed touch conditions to conditions where no touch was observed, we found significant activity increases in left (contralateral) S1 as a main effect. This effect was not only found at the group level but also in almost all individual participants. Significant activity changes in right (ipsilateral) S1 were only found when the group level statistics were analyzed at uncorrected thresholds. The results of the present study show that observing touch to fingers of a right hand, therefore, clearly evokes activity increases in left posterior S1.

The finding that touch observation can elicit activity increases in S1 is in accordance with a growing body of evidence suggesting the independence of S1 activity from direct somatosensory input (Chen et al. 2003; Yoo et al. 2003; Driver and Noesselt 2008; Meehan et al. 2009; Wood et al. 2010). Specifically touch observation has, in a number of fMRI studies, been shown to trigger profound activity increases in S1 (Blakemore et al. 2005; Ebisch et al. 2008; Kuehn et al. 2012; Schaefer et al. 2005, 2006, 2009). A previous study also indicated that in particular posterior rather than anterior contralateral S1 responds to the observation of tactile events (Kuehn et al. 2012). This appears to contrast findings reported in an fMRI study by Schaefer et al. (2009). Participants in that study also observed short video sequences where hands were either touched or not touched by paintbrushes. Whereas, in accordance with the present results and those previously reported, touch observation induced activity increases in posterior contralateral S1, the authors also reported responsivity of anterior S1, specifically when participants looked at a hand presented in a first-person viewing perspective. A recent study re-investigating this topic using 7 T fMRI (Kuehn et al. 2012) yielded divergent findings. In that study, anterior S1 did not show significant activity changes, neither as a main effect, nor when first-person and third-person viewing perspectives were directly compared. Kuehn et al. (2012) argued that the lower spatial resolution of the data used by Schaefer et al. (2009), in terms of voxel size and smoothing, may have accounted for the divergent findings. Although an involvement of anterior S1 cannot be excluded, it seems that the major responsivity of S1 during touch observation stems from its posterior parts. This is also in accordance with the recently formulated hypothesis that posterior S1 in particular is open to social influences, for example during action observation (Keysers et al. 2010).

The high connectivity between posterior S1 and visual input areas in the parietal cortex, some of which are known to contain bimodal visuo-tactile neurons (Duhamel et al. 1998; Ishida et al. 2010; Lewis and Van Essen 2000; Maunsell and van Essen 1983; Pons and Kaas 1986; Rozzi et al. 2006) and to show bimodal activation pattern in humans (Sereno and Huang 2006), can serve to explain this greater influence of vision on activity changes in the posterior rather than the anterior part of S1. Anterior S1 is more strongly connected to the thalamus than posterior S1 (Kaas 2008; Nelson and Kaas 1981); therefore, we assume that the thalamus did not strongly contribute to the S1 activity observed in the present study. A dichotomic division of S1 into posterior S1, showing pronounced reactivity to visual input (e.g., during observed touch) and anterior S1, which may still be regarded as a unisensory brain area mainly driven by bottom-up somatosensory input, has been suggested previously (Keysers et al. 2010; Kuehn et al. 2012), and is supported by our results.

Another important question is whether the activity changes in posterior S1 found in our study were triggered by touch observation, or resulted from preparatory motor responses or mental imaginary of action. Given the role of area 2 in proprioception (Hsiao and Bensmaia 2008), and the involvement of S1 in motor preparation (Kawashima et al. 1994), such an explanation cannot a priori be excluded. However, for the present findings, this explanation is highly unlikely. Activity changes in S1 were strongly lateralized to left S1 (contralateral to the observed touch events), whereas right S1 (contralateral to the motor response) showed only sub-threshold activity. In addition, we did not find any activity increases in left or right M1 during touch observation, which would be expected if one assumed an involvement of motor preparation (Kawashima et al. 1994) or motor imaginary (Dushanova and Donoghue 2010). We are, therefore, confident that the S1 activity reported in the present study is due to touch observation rather than preparatory motor activity or motor imaginary.

### Topography of S1 activity during touch observation

In order to describe the functional architecture of posterior S1 during touch observation, we first looked at whether S1 activity during observed and physically perceived touch showed a regional overlap. Any overlap would indicate a resonance response (Hogeveen and Obhi 2012; Landmann et al. 2011; Virji-Babul et al. 2012) within S1 between physically perceived and observed touch. Such resonance responses have often been described for the motor system (Buccino et al. 2001; Mukamel et al. 2010; see Caspers et al. 2010 and Gazzola and Keysers 2009 for an overview), the insula (Corradi-Dell’Acqua et al. 2011; Singer et al. 2004; see Bernhardt and Singer 2012 and Lamm et al. 2011 for an overview), S2 (Keysers et al. 2004), and also for S1 (Blakemore et al. 2005; Ebisch et al. 2008; Schaefer et al. 2009). However, so far, they have not been characterized with such a high sensitivity and high spatial specificity as offered by the design of the present study. Whereas previous studies indicated spatial specificity of S1 activity when touch to different body areas, such as the face and neck, was observed (Blakemore et al. 2005), or showed that observing hand touch elicited specific activity increases in the hand area of S1 (Kuehn et al. 2012; Schaefer et al. 2009), the present study indicates a spatially specific resonance response at the level of the single finger. More precisely, observing touch to the index finger overlapped with activity changes during physically experiencing touching of the index finger, and observing touch to the middle finger overlapped with activity changes during physically experiencing touching of the middle finger. Interestingly, whereas this resonance response seemed relatively specific for the index finger (i.e., S1 responses to observing touch to the index finger significantly overlapped with those to physical touching of the index finger, but did not significantly overlap with those to physical touching of the middle finger), this specificity was not present for observing touch to the middle finger. Here, the same significance level was reached irrespectively of whether the contrast of observing touch to the middle finger was masked with physical touching of the index finger or physical touching of the middle finger. In addition, the responsivity of S1 was generally larger when touching to the index finger was observed compared with when touching to the middle finger was observed. These results indicate that observing touch to the index finger leads to higher and spatially more specific responses in S1 compared to observing touch to the middle finger. These results could be explained by the generally enhanced use of the index finger compared to the middle finger, for example during the so called precision grip (Napier 1956). In studies on the motor system, greater experience in a certain motor behavior has been shown to lead to increased responses in the motor system not only during action performance (Karni et al. 1995, 1998) but also during action observation (Calvo-Merino et al. 2005; Cross et al. 2006). In addition, a more precise response of the action observation network has also been assumed for participants that have more experience with the observed actions (Cross et al. 2012; Diersch et al. 2012). One may therefore argue that the results of the present study indicate a similar relation in the somatosensory system. Increased tactile experience of a certain body area, such as the index finger, that has been shown to relate to increased S1 activity (Braun et al. 2000; Pleger et al. 2003) and better discrimination abilities (Braun et al. 2000; Ragert et al. 2003; Schweizer et al. 2001) during physical touch perception, may also cause a stronger and more precise representation during touch observation.

It is important to note, however, that the present data do now allow a direct comparison between touch observation and physical touch perception. Whereas in the observed touch videos, different paintbrushes were used for tactile stimulation, and a roughness task had to be solved, in the physical touch experiment, tactile stimulation was applied passively, and the same paintbrushes were used for tactile stimulation. The experimental set-ups therefore differ, and do not allow direct comparison of S1 RFs as evoked by visual and physical touch perception. Future studies should use completely analogue designs with respect to stimulus characteristics and attention requirements in order to compare the overlap between RFs in both conditions more precisely. Only such a design would finally allow conclusions to be drawn about the specificity of the activity overlap between physical touch perception and touch observation.

A second main aspect that characterizes S1 topography during touch observation is the topographical arrangement of the evoked activity changes. Our results show that activity changes in S1 during touch observation to the index finger were partly distinct, and located more lateral, more anterior, and more inferior than activity changes in S1 during touch observation to the middle finger. This topographical alignment of index and middle finger RFs follows exactly the same pattern as has classically been described for physical touch (Nelson and Chen 2008), and as has also been found in the present study. This indicates a surprisingly precise representation of observed touch events in S1, and assumes a precision down to the level of the single finger. Should further studies manifest this finding, this would offer another parallel to the action system. Also, observing motor movements of specific body parts has been shown to elicit somatotopically precise representations in the premotor cortex (Buccino et al. 2004; Wheaton et al. 2004); the present study assumes a similarly spatially specific and precise representation of observed human touch.

Taken together, our results indicate that observing touch to single fingers does not simply activate the hand area in S1, but activates parts in S1 that are topographically precise. The spatial arrangement of S1 activity seems therefore highly similar during physically perceived and observed touch, which leads to the suggestion that not only action events but also tactile events can be shared between the observed person and the observer (Bufalari et al. 2007).

### Suppressive interactions during touch observation

In the present study, the functional architecture in S1 was additionally characterized by looking at suppressive interactions between adjacently activated cortical RFs. Suppressive interactions in S1 have often been characterized by measuring the relative shrinkage of index and middle finger RFs when both are activated simultaneously, compared to when they are activated alone (Gandevia et al. 1983; Ruben et al. 2006). Using this approach in the present study, we found suppressive interactions mainly in posterior parts of contralateral S1, slightly extending to anterior S1. This confirms previous studies that found greater suppressive interactions during touch perception in posterior contralateral S1 (Friedman et al. 2008; Ruben et al. 2006; Sur 1980; Sripati et al. 2006), which may indicate an increasing convergence of somatosensory input from anterior to posterior sites of S1 (Ruben et al. 2006). Also the mean interaction ratio found in the present study (38 %) was similar to that which has been described previously (Biermann et al. 1998; Gandevia et al. 1983; Ruben et al. 2006). These comparable results between the present and previous attempts to characterize suppressive interactions in S1 confirm that the present approach was, in principle, suitable to characterize this phenomenon.

This is important given that analogue contrasts were used to characterize suppressive interactions during touch observation. This characterization was attempted for the first time in the present study. Here, we looked at whether suppressive interactions in S1 would similarly occur when touch to two fingers, compared to two single fingers separately, was not physically experienced but merely observed. Our data indicate that suppressive interactions in S1 may also occur during touch observation. More precisely, we found that observing touch to two fingers elicited decreased activity levels in S1 compared to observing touch to two single fingers separately, an effect that was specific for the areas in S1 where observed touch to single fingers separately elicited effects. Spatially, the effect was restricted to contralateral posterior S1, which was expected given that touch observation particularly activated posterior parts of contralateral S1. Importantly, suppressive interactions were also found in voxels that did not overlap between index and middle finger RFs, making vascular ceiling or saturation effects an unlikely explanation for the observed effects (Beauchamp et al. 2004; Gardner and Costanzo 1980).

It is important to note, however, that while the principle way of characterizing suppressive interactions during visual and physical touch perception in the present study was similar, the results of these two analyses should not be compared directly. During physical touch perception, participants lay in the scanner with their eyes closed, while they actively solved a roughness discrimination task during touch observation. Because previous research has evidenced an influence of attention on suppressive interactions in S1 (Braun et al. 2002), the suppressive interaction effect in both conditions is not directly comparable because attentional demands varied between both experiments. Secondly, there was a difference in control conditions. During touch observation, S1 activity changes were compared to a control condition (i.e., where participants saw hands which were not being touched), whereas in the physical touch condition, no such control condition was present (i.e., physical touch perception was compared to a rest condition). Given that merely looking at hands may influence S1 activity (Fiorio and Haggard 2005; Longo et al. 2011) and the degree of suppressive interactions in S1 (Cardini et al. 2011), one should avoid comparing the degree of suppressive interactions between physical and visual touch perception in the present study directly.

The indicated existence of suppressive interactions in S1 during touch observation can be embedded into the results from recent studies that assign S1 a specific and highly flexible role during touch and action observation (Avenanti et al. 2005; Bolognini et al. 2011; Bufalari et al. 2007; Caspers et al. 2010; Keysers et al. 2010; Meyer et al. 2011). For instance, using multivariate pattern analysis, it has been shown that activity patterns in S1 are separable when haptic exploration of different everyday objects is observed (Meyer et al. 2011). Such variable and spatially specific activity changes in S1 could be regarded as an indication of the existence of inhibitory regulatory mechanisms that modulate S1 activity during touch observation. More direct evidence that vision can influence the degree of suppressive interactions in S1 during physical touch perception is offered by studies investigating somatosensory evoked potentials (SEPs) in different viewing conditions. Here, it has been assumed that looking at a body while receiving tactile stimulation increases suppressive interactions in S1 (Cardini et al. 2011; Gillmeister et al. 2010), which has been related to RF sharpening in this condition (Cardini et al. 2011; Haggard et al. 2007). Whereas the results of the present study are, therefore, in accordance with previous investigations, they are novel because they target the mechanism of suppressive interactions during touch observation for the first time directly.

### The relation between suppressive interactions and behavioral performance

During physical touch perception, suppressive interactions are assumed to positively relate to perceived stimulus contrast (Braun et al. 2002; Cardini et al. 2011; Puts et al. 2011). We therefore hypothesized that, if suppressive interactions during physical and observed touch share a mechanistic basis, such a relation to the ability to discriminate tactile stimulus features should also occur during touch observation. This analysis, however, was hampered by the very small sample size available to calculate this correlation (*n* = 9). However, when looking at the correlation, the degree of suppressive interactions during observed touch related positively to performance levels to discriminate roughness levels of paintbrushes by sight. Although this relation clearly needs further exploration in future studies, it indicates a powerful message: the degree of suppressive interactions in S1 during observed touch may determine the precision with which observed tactile events can be decoded by the observer. This is particularly interesting because, so far, signal decreases in S1 during touch observation are mostly assumed to indicate a lower resonance response, and are, thus, interpreted as evidence for lower degrees of inner simulation (Blakemore et al. 2005; Ebisch et al. 2008; Kuehn et al. 2012). Given the results of the present study, this view may be rather one-dimensional. Signal decreases, at least when they can clearly be assigned to the occurrence of suppressive interactions, may indicate a more precise and less noisy, rather than a weaker, stimulus representation. This similarly holds for action observation. A recent study showed that the BOLD response in the action observation network, which is classically assumed to increase during observed actions that are more familiar (Buccino et al. 2004; Calvo-Merino et al. 2005; Cross et al. 2006), does not increase when more familiar actions compared to less familiar actions are observed (Cross et al. 2011). Given our framework, one may speculate that the decreases in the BOLD signal indicate a more precise representation of the observed familiar movements. Future studies should, therefore, take decreases of the BOLD signal into account when investigating the role of S1, or other brain areas, in the realm of social cognition.

## Conclusions

Taken together, the results from our study provide strong evidence that posterior contralateral S1 is active during touch observation, and that these activity changes overlap with those elicited by physical touch experience. In addition, our results indicate that touch observation to single fingers elicits partly distinct and topographically precise single finger representations in S1, which show similar dynamic interactions as they do during physical touch perception. Although this study only provides a first step to understanding the functional architecture of S1 in a social context, it critically emphasizes the importance of taking fine-grained architectonic details into account when describing the role of S1 in social cognition.

## Electronic supplementary material

Below is the link to the electronic supplementary material.

Supplementary material 1 (DOC 29 kb)

Supplementary material 2 (DOC 66 kb)

Supplementary material 3 (DOC 423 kb)
