# Supplementary material for: The functional architecture of S1 during touch observation described with 7 T fMRI
Source: Brain Struct Funct. 2013 Jan 3;219(1):119–40. doi: 10.1007/s00429-012-0489-z (PMC3889700; doi:10.1007/s00429-012-0489-z)
Supplement: Supplementary file 1 — Supplementary material 1 (DOC 29 kb) [file 429_2012_489_MOESM1_ESM.doc]

**Online Resource 1:** Behavioral pre-experiment: Roughness discrimination of paintbrushes

Article: The functional architecture of S1 during touch observation described with 7 Tesla fMRI

Brain Structure and Function

Esther Kuehn^1^, Karsten Mueller^1^, Robert Turner^1^, Simone Schütz-Bosbach^1^

^1^Max Planck Institute for Human Cognitive and Brain Sciences, Leipzig, Germany

Email corresponding author: [ekuehn@cbs.mpg.de](mailto:ekuehn@cbs.mpg.de)

Rationale

In a behavioral pre-experiment, we tested whether the four paintbrush pairs displayed in the video clips for the observed touch experiment (see Stimuli section main document) could be reliably distinguished in their roughness levels by physical touch.

Participants

Nine healthy volunteers (5 females) participated in this experiment. None of them participated in the two fMRI studies. They were all right-handed (mean handedness score: 92.9, Oldfield 1971) and between 20 and 30 years of age (mean age: 25.5 years ± 2.7 [*SD*)). They were paid for their attendance and informed consent was obtained from all participants.

Experimental Design

During the experiment, participants sat on a chair and looked at a fixation cross in front of them. They comfortably placed their right hand on a table next to them. They were told to completely relax their hand and fingers throughout the experiment. Between the participant and the table, a paper wall was mounted which prevented participants from seeing their hand, the paintbrushes, or the experimenter. The experimenter sat on the other side of the paper wall and applied tactile stimulation to the participants’ right index fingers using different paintbrushes. The same experimenter also applied tactile stimulation during the video recordings (see Stimuli section main document) and was trained to always apply tactile stimulation with equal strength and pressure. Analogous to the video recordings, tactile stimulation was applied to the index finger via two continuous strokes using the same paintbrushes (3 s per stroke, 6 seconds in total). An auditory signal indicated to the experimenter the temporal sequence of stimulation. After 4 s pause, a second paintbrush was used to apply tactile stimulation to the same finger in the same temporal sequence and with equal pressure. Three s after the second stimulation was finished, a tone motivated the participants to respond via left hand button press which paintbrush they felt was rougher. Half of the participants pressed the button with their left index finger when they thought the first paintbrush was rougher and with their left middle finger when they thought the second was rougher, the other half responded vice versa. Participants were required to answer within a two-seconds time window. After a pause of 7 s the next trial began. Altogether, four different paintbrush pairs were used during tactile stimulation trials ([1] versus [4], [2] versus [5], [1] versus [2], and [3] versus [5]; for specifications of the paintbrushes see Stimuli section main document). The experiment consisted of 40 trials in total, thus each paintbrush pair was used for stimulation 10 times. In half of the trials, the smoother paintbrush was used first, in the other half the rougher paintbrush was used first. The trial order was randomized and was indicated to the experimenter via a screen in front of him.

Analyses and Results

To see whether participants would be able to reliably differentiate between roughness levels of the different paintbrushes, we calculated the mean percentage of correct responses given for each paintbrush pair and tested them against chance using one-sample *t*-tests (two-tailed, Holm-Bonferroni corrected). Our results showed that all participants could detect roughness differences between the two paintbrushes of each paintbrush pair with high levels of accuracy. The percentage of correct performances significantly differed from chance in all experimental conditions ([1] versus [2]: 100.0% correct ± 0.0 (*SD*); [3] versus [5]: 85.6% correct ± 19.4 (*SD*), t_8_ = 5.49, *p* < 0.001; [1] versus [4]: 93.3% correct ± 7.1 (*SD*), t_8_ = 18.38, *p* < 0.0005; [2] versus [5]: 70.0% correct ± 21.2 (*SD*), t_8_ = 2.83, *p* < 0.02).
