# Supplementary material for: The functional architecture of S1 during touch observation described with 7 T fMRI
Source: Brain Struct Funct. 2013 Jan 3;219(1):119–40. doi: 10.1007/s00429-012-0489-z (PMC3889700; doi:10.1007/s00429-012-0489-z)
Supplement: Supplementary file 2 — Supplementary material 2 (DOC 66 kb) [file 429_2012_489_MOESM2_ESM.doc]

**Online Resource 2:** List of sub-threshold activity changes for main contrasts

Article: The functional architecture of S1 during touch observation described with 7 Tesla fMRI

Brain Structure and Function

Esther Kuehn^1^, Karsten Mueller^1^, Robert Turner^1^, Simone Schütz-Bosbach^1^

^1^Max Planck Institute for Human Cognitive and Brain Sciences, Leipzig, Germany

Email corresponding author: [ekuehn@cbs.mpg.de](mailto:ekuehn@cbs.mpg.de)

|  | **Contrast** | **Peak Area** | **MNI location (*x*, *y*, *z*)** | **Peak *t*-value** | **No of voxels** |
| --- | --- | --- | --- | --- | --- |
| **Physical Touch** | MF touch + IF touch + BF touch - rest | L IPC  L Area 3b  L Area 3b  R Area 3b | -55 -24 40  -48 -21 57  -42 -28 42  53 -18 34 | 8.31  4.30  4.29  4.05 | 685  8  6  9 |
|  | MF touch - rest | L IPC  L Area 2 | -55 -24 40  -44 -33 57 | 7.33  6.78 | 185  215 |
|  |  |  |  |  |  |
|  | IF touch - rest | L Area 3b  R Area 3b  R Area 1 | -37 -39 63  54 -16 32  65 -14 32 | 9.03  4.50  4.41 | 562  28  8 |
|  | BF touch - rest | L IPC | -56 -22 42 | 9.37 | 991 |
|  | SI physical touch | L IPC | -54 -26 39 | 7.47 | 123 |
| **Observed Touch** | obs. touch MF + obs. touch IF + obs. touch BF – no-touch | L Area 2  L Area 2  L Area 2  L SPL  R Area 2  R Area 2  R Area 3b  R Area 1  R Area 2 | -37 -44 54  -55 -24 44  -26 -51 57  -26 -54 66  17 -50 62  32 -48 66  30 -40 56  64 -15 39  23 -46 56 | 4.35  4.04  5.47  5.04  5.31  4.81  4.33  4.17  3.93 | 245  179  19  6  5  33  25  6  8 |
|  |  |  |  |  |  |
|  | obs. touch MF - no-touch | L SPL  L Area 2  R Area 2  R Area 3b  L Area 2  L IPC | -26 -54 66  -26 -51 57  24 -44 54  46 -24 40  -54 -27 45  -60 -18 36 | 5.02  5.00  4.92  4.68  3.45  3.29 | 6  17  36  8  19  9 |
|  |  |  |  |  |  |
|  |  |  |  |  |  |
|  |  |  |  |  |  |
|  | obs. touch IF - no-touch | L IPC  L Area 2  R Area 2  L Area 2  L IPC  R Area 2 | -58 -21 40  -36 -44 57  22 -48 60  -26 -51 57  -36 -36 44  35 -50 66 | 6.05  5.75  4.70  4.58  4.49  4.47 | 139  104  14  6  13  5 |
|  | obs. touch BF - no-touch | L Area 2  R Area 2 | -34 -44 57  32 -48 66 | 6.27  6.02 | 127  57 |
|  |  | L Area 2  L SPL  L Area 2  R Area 3b  R Area 1  R Area 2 | -54 -24 44  -26 -54 66  -25 -50 60  58 -16 33  62 -15 40  29 -40 56 | 5.56  5.34  5.05  4.89  4.63  4.23 | 128  6  18  46  15  16 |
|  | SI observed touch | L Area 2  L IPC | -55 -26 46  -36 -36 44 | 4.54  4.51 | 8  5 |
|  |  |  |  |  |  |
|  | SI observed touch self | L Area 2  L Area 2 | -49 -36 57  -42 -40 62 | 4.93  4.62 | 9  15 |
| **Observed Touch**  **∩ Physical Touch** | obs. touch MF + obs. touch IF + obs. touch BF – no-touch **∩** MF touch + IF touch + BF touch - rest | L Area 2  L Area 3b | -55 -24 44  -38 -40 60 | 5.70  4.46 | 162  19 |
|  | obs. touch MF - no-touch **∩**  MF touch – rest | L Area 2  L OP4 | -54 -27 45  -61 -16 33 | 4.45  4.15 | 15  10 |
|  | obs. touch IF - no-touch **∩**  IF touch - rest | L IPC | -58 -21 40 | 6.05 | 123 |
|  | obs. touch BF - no-touch **∩**  BF touch - rest | L Area 2 | -54 -24 44 | 5.56 | 114 |
| *Note*: Listed clusters contain voxels thresholded at *p* < 0.001 that contain a minimum of 5 voxels; obs. = observed, MF = middle finger, IF = index finger, BF = both fingers, SI = suppressive interactions, IPC = inferior parietal cortex, SPL = superior parietal lobule, OP = parietal operculum. | | | | | |
