# Supplementary material for: The functional architecture of S1 during touch observation described with 7 T fMRI
Source: Brain Struct Funct. 2013 Jan 3;219(1):119–40. doi: 10.1007/s00429-012-0489-z (PMC3889700; doi:10.1007/s00429-012-0489-z)
Supplement: Supplementary file 3 — Supplementary material 3 (DOC 423 kb) [file 429_2012_489_MOESM3_ESM.doc]

**Online Resource 3:** Relation between suppressive interactions in S1 and behavioral performance in visual roughness discrimination task

Article: The functional architecture of S1 during touch observation described with 7 Tesla fMRI

Brain Structure and Function

Esther Kuehn^1^, Karsten Mueller^1^, Robert Turner^1^, Simone Schütz-Bosbach^1^

^1^Max Planck Institute for Human Cognitive and Brain Sciences, Leipzig, Germany

Email corresponding author: [ekuehn@cbs.mpg.de](mailto:ekuehn@cbs.mpg.de)


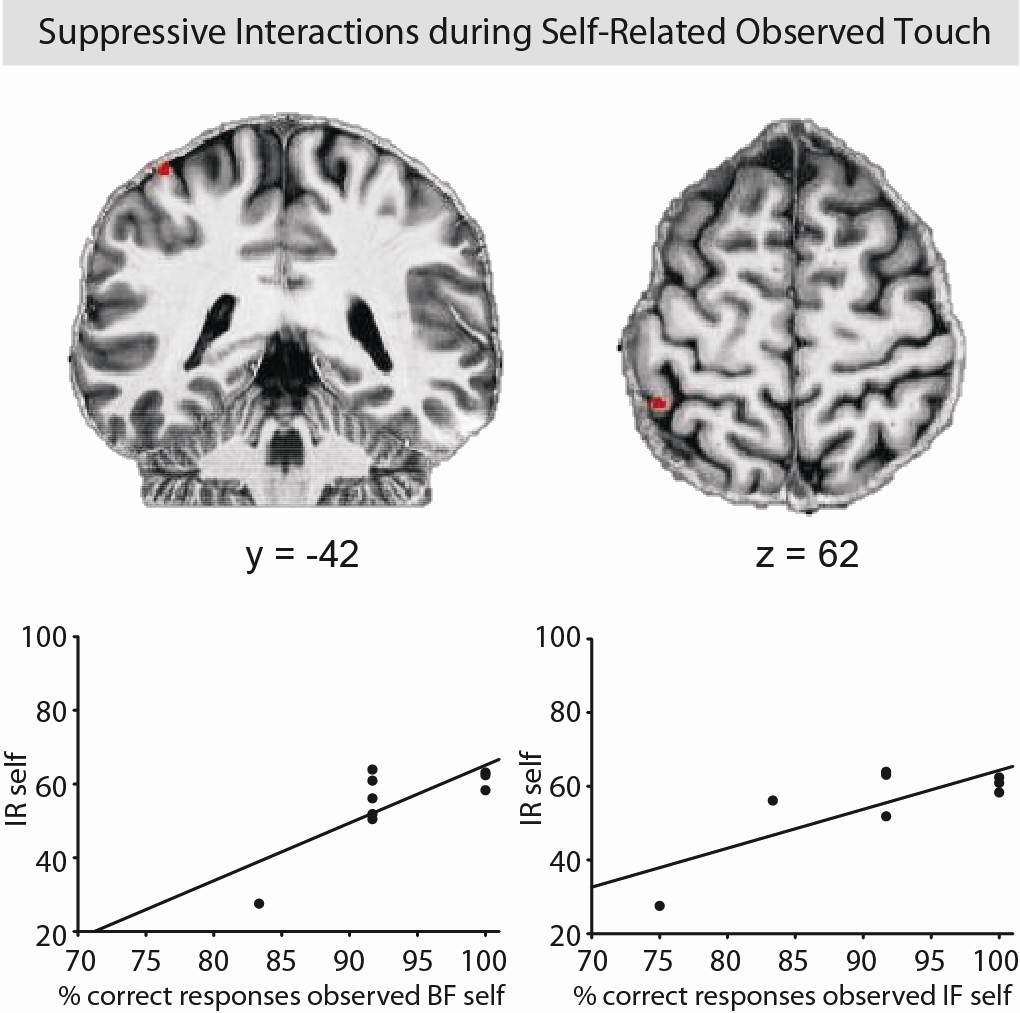


Relation between suppressive interaction effect when touch to a self-hand is observed and performance levels in a visual roughness discrimination task. Shown are positive correlations between the interaction ratio (IR) during self-related observed touch and the percentage of correct responses in the visual roughness discrimination task when self-related touch to both fingers (BF) and the index finger (IF) was observed (*r* = 0.78 for IF, and *r* = 0.76 for BF self, *p* < 0.05, Pearson-correlation, two-tailed). There was no such relation between the individual IRs during observed touch and percentage of accuracy across conditions. Note, however, that the number of subjects to calculate this correlation (*n* = 9) was too small, such that this relation has to be replicated and verified by future studies.
